# Up-regulation expression and prognostic significance of Syntaxin4 in kidney renal clear cell carcinoma

**DOI:** 10.1186/s12885-021-08736-1

**Published:** 2021-09-06

**Authors:** Lishan He, Huiming Jiang, Zhenqiang Lai, Zhixiong Zhong, Zhanqin Huang

**Affiliations:** 1 Department of Clinical Pharmacy, Meizhou People’s Hospital (Huangtang Hospital), Huangtang Road 63#, Meijiang District, Meizhou, People’s Republic of China 514031; 2 Department of Urology, Meizhou People’s Hospital (Huangtang Hospital), Meizhou, 514031 China; 3 Pharmacy Intravenous Admixture Services, Meizhou People’s Hospital (Huangtang Hospital), Meizhou, 514031 China; 4 Center for Cardiovascular Diseases, Meizhou People’s Hospital (Huangtang Hospital), Huangtang Road 63#, Meijiang District, Meizhou, People’s Republic of China 514031; 5 Department of Pharmacology, Shantou University Medical College, Xinling Road 22#, Shantou, People’s Republic of China 515041

**Keywords:** Syntaxin4, Kidney renal clear cell carcinoma, Endo/exocytosis, Cell invasion, Prognostic

## Abstract

**Background:**

Syntaxin4 (STX4) gene encodes the protein STX4, a member of soluble N-ethylmaleimide-sensitive factor attachment protein receptors protein, playing a vital role in cell invadopodium formation and invasion, which is associated with the malignant progression of various human cancers. However, the expression and prognostic significance of STX4 in kidney renal clear cell carcinoma (KIRC) remain to be investigated.

**Methods:**

In this study, we collected the mRNA expression of STX4 in 535 KIRC patients from The Cancer Genome Atlasthrough the University of California Santa Cruz Xena database platform. Then we explored the expression of STX4 in KIRC, and the relationship with clinicopathological characteristics and prognostic value. Gene Ontology and Kyoto Encyclopedia of Genes and Genomes function enrichment analyses were used to explore the potential mechanism of STX4 in KIRC. qRT-PCR analysis was performed toverify the above results with real world tissue specimens.

**Results:**

The results indicated that STX4 was up-expressed in KIRC, and were associated with higher histological grade, advanced stage, and poorer prognosis. Moreover, elevated STX4 expression is an independent risk factor for KIRC. qRT-PCR analysis showed that STX4 was significantly elevated in 10 paired of KIRC samples compared to normal samples. Functional enrichment analysis indicated that endo/exocytosis, autophagy, mTOR signaling pathway, and NOD-like receptor signaling pathway were enriched.

**Conclusions:**

In summary, STX4 is constantly up-expressed in KIRC tissues, associated with a poor prognosis. We suggest that it can be an effective biomarker for the prognosis of KIRC and may be a novel therapeutic target in KIRC.

## Background

Kidney renal clear cell carcinoma (KIRC) is the main histological subtype of the renal cell carcinoma (RCC), accounting for 80–90% of patients [1]. KIRC was one of the ten leading cancer types for the estimated new cancer cases and deaths in the United States, and it had caused about 14,770 new deaths according to cancer statistics data in 2019 [2]. More than half of KIRC patients are a symptomless and diagnosed incidentally on imaging [3]. Although nephrectomy or targeted therapies had been implementation, approximately 30% of patients with localized tumor eventually develop metastases and the 5-year survival rate for patients with metastatic is less than 10% [4, 5]. The TNM stage is usually used as risk predictors for KIRC [6], but the outcomes for KIRC were heterogeneous in various aspects including clinicopathological, molecular, and cellular heterogeneity. Therefore, it is urgent to study the carcinogenesis and progression of KIRC and explore new useful molecular markers for prognosis.

Metastasis is a complex multicellular process that dependents on tumor cell invasion through the extracellular matrix, a supportive scaffold that acts to compartmentalize tissues [7]. Membrane trafficking of cellular cargo mediated in part by soluble N-ethylmaleimide-sensitive factor attachment protein receptors (SNAREs), a family of membrane proteins that form complexes bridging apposed membranes and allowing membrane fusion [8]. Syntaxin4 (STX4) is one of SNAREs proteins implicated in the trafficking of membrane type-1 matrix metalloproteinase (MT1-MMP) to the plasma membrane [9]. Recent research has shown that STX4 mediates invadopodium formation and tumor cell invasion [10]. However, it is unclear whether STX4 involved in the metastasis of KIRC. In this study, we explored and identified the STX4 associated with survival in patients with KIRC. We found that the SNAREs protein STX4 was positively correlated with malignant clinicopathological characteristics and was significantly related to overall survival (OS) in patients with KIRC. Most importantly, elevated STX4 expression is an independent risk factor for KIRC.

## Methods

### Data extraction and identification of prognostic STX4 in KIRC

The study is approved by Medical Ethics Committee of Meizhou People’s Hospital (2020-CY-06) and in accordance with the ethical standards of the institutional and/or national research committee and with the 1964 Helsinki declaration and its later amendments or comparable ethical standards. We collected the mRNA expression of STX4 and clinical data of 535 KIRC patients from The Cancer Genome Atlas (TCGA) through the University of California Santa Cruz Xena database platform (https://xena.ucsc.edu/). Among them, 531 KIRC patients had information regarding survival. We matched the patients’ clinical information and STX4 mRNA expression data. The different expression of STX4 was compared between 535 KIRC samples and 72 normal kidney samples. The correlation between the expression level of STX4 and clinicopathological characteristics in KIRC was also assessed. Kaplan–Meier (KM) survival curves was used for prognosis analysis using the R packages “survival” and “survminer”, Log-rank test *P* value< 0.05 was chosen to be significantly different. Finally, we validated the results using the pan-cancer data from TCGA (32 other types of cancer) and qRT-PCR results of 10 paired of KIRC and normal real-world samples as internal and external validations, respectively.

### Quantitative reverse transcription polymerase chain reaction (qRT-PCR)

To further validate the RNA-sequencing data obtained from TCGA, qRT-PCR analysis was performed to validate the expression of STX4 in 10 paired of KIRC and normal samples. We collected KIRC samples and paired adjacent normal samples from 10 patients who underwent nephrectomies or partial nephrectomies at Meizhou People’s Hospital between 2019 and 2020. Informed consent was obtained from all patients. We extracted total RNA using the TRIzol™ reagent (Waltham, Massachusetts, USA). First-strand complementary DNA was synthesized equal amounts of total RNA (4 μg) using the PrimeScript RT reagent kit (Takara Bio, Inc., Dalian, China) according to the manufacturer’s instructions. Analyzed by The SYBR Green PCR kit (Takara Bio, Inc., Dalian, China) incorporation in PCR reactions involving specific primers and performed in the ABI 7500 fluorescent quantitative PCR system (Applied Biosystems Inc., Foster City, CA, USA). Glyceraldehyde 3-phosphate dehydrogenase (GAPDH) was used as the internal control. Thespecific primer sequences were as follows: STX4 (forward: CTGTCCCAGCAATTCGTGGAG; reverse: CCCAGCATTGGTGATCTTCAG), and GAPDH (forward: ATGACATCAAGAAGGTGGTG; reverse: CATACCAGGAAATGA GCTTG). The expression level was also calculated using the 2^–ΔΔCt^ method.

### Functional and pathway enrichment analyses

To identify biological functions in STX4 gene set, we carried out a Gene Ontology (GO) classification, which included the following categories: biological process, cellular component, and molecular functions. We firstly explored the co-expressed genes with the STX4 (correlation coefficient r > 0.4, *P* <  0.001). Then we used the entire co-expressed genes matrix after pre-processing as a background, and performed GO functional enrichment analysis at online tools (http://kobas.cbi.pku.edu.cn/) [11]. We explored the Kyoto Encyclopedia of Genes and Genomes (KEGG) analysis with the same method.

### Statistics

All data processing and statistical analysis were performed using R (version 3.6.1; The R Foundation for Statistical Computing, Vienna, Austria), Strawberry Perl (version 5.30.1.1; http://strawberryperl.com/), and Statistical Pack/ age for Social Sciences (version 25.0; IBM, Armonk, New York, USA). Analysis of variance or *t*-test was used to compare the gene expression level among different subgroups. The differences were considered significant when *P*<0.05.

## Results

### STX4 expression was significantly up-regulated in KIRC

**Fig. 1:**
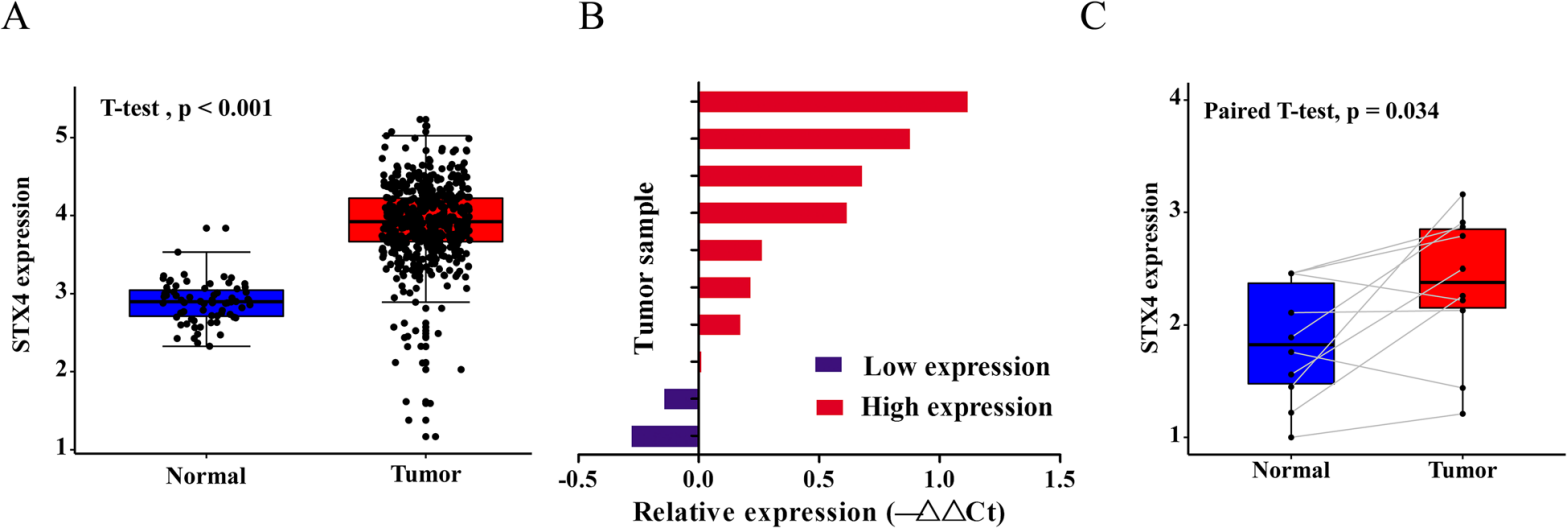
Expression of STX4 mRNA in human KIRC. (**A**) STX4 transcription level was significantly higher in 535 KIRC tissue samples than in 72 normal tissue samples. (**B**) STX4 was up-regulated in 8 of 10 KIRC tissue samples. (**C**) Compared with normal tissue samples, STX4 was significantly up-regulated in tumor samples of 10 paired samples

After matching the patients’ clinical information and STX4 gene expression data, 535 patients with KIRC were enrolled in the study. The 535 patients’ clinicopathological features are shown in Table 1. After analyzing the expression levels of STX4 in 535 KIRC samples and 72 normal kidney samples, we found that the expression of STX4 in KIRC tissues was obviously higher compared with normal tissues (*P*<0.05) (Fig. 1A). To validate STX4 mRNA expression in KIRC tissues, we performed qRT-PCR in 10 paired tumor and normal samples. Compared with normal tissues, the expression of STX4 in KIRC tissues was significantly elevated by qRT-PCR result (*P*<0.05) (Fig. 1B,C).

**Table 1:** Correlations between the expression of STX4 and clinicopathologic characteristics in KIRC

Characteristic	n (%)	Expression of STX4 (%)		*P*-value
		High	Low	
Total	535 (100)	267 (49.91)	268 (50.09)	
Age				0.437
ZA ≤ 60 years	267 (49.91)	138 (51.7)	129 (48.1)	
> 60 years	268 (50.09)	129 (48.3)	139 (51.9)	
Gender				0.277
Female	186 (34.77)	99 (37.1)	87 (32.5)	
Male	349 (65.23)	168 (62.9)	181 (67.5)	
Cancer status				0.003
Tumor free	336 (62.8)	155 (58.1)	181 (67.5)	
With tumor	148 (27.66)	90 (33.7)	58 (21.6)	
Unknow	51 (9.53)	22 (8.2)	29 (10.8)	
Race				0.191
White	463 (86.54)	226 (84.6)	237 (88.4)	
Asian	8 (1.5)	4 (1.5)	4 (1.5)	
Black	57 (10.65)	35 (13.1)	22 (8.2)	
Unknow	7 (1.31)	2 (0.7)	5 (1.9)	
Grade				< 0.001
G1	14 (2.62)	8 (3.0)	6 (2.2)	
G2	231 (43.18)	91 (34.1)	140 (52.2)	
G3	207 (38.69)	119 (44.6)	88 (32.8)	
G4	75 (14.02)	48 (18.0)	27 (10.1)	
Unknow	8 (1.5)	1 (0.4)	7 (2.6)	
T stage				< 0.001
T1	275 (51.4)	164 (61.4)	111 (41.4)	
T2	70 (13.08)	32 (12.0)	38 (14.2)	
T3	179 (33.46)	70 (26.2)	109 (40.7)	
T4	11 (2.06)	1 (0.4)	10 (3.7)	
N stage				0.616
N0	240 (44.86)	118 (44.2)	122 (45.5)	
N1	16 (2.99)	9 (3.4)	7 (2.6)	
Unknow	279 (52.15)	140 (52.4)	139 (51.9)	
M stage				< 0.001
M0	424 (79.25)	188 (70.4)	236 (88.1)	
M1	78 (14.58)	52 (19.5)	26 (9.7)	
Unknow	33 (6.17)	27 (10.1)	6 (2.2)	
AJCC stage				< 0.001
Stage I	269 (50.28)	110 (41.2)	159 (59.3)	
Stage II	58 (10.84)	29 (10.9)	29 (10.8)	
Stage III	123 (22.99)	72 (27.0)	51 (19.0)	
Stage IV	82 (15.33)	54 (20.2)	28 (10.4)	
Unknow	3 (0.56)	2 (0.7)	1 (0.4)	

*KIRC* Kidney renal clear cell carcinoma, *AJCC* American Joint Committee on Cancer

### Up-expression of STX4 predicts poor prognosis of KIRC

**Fig. 2:**
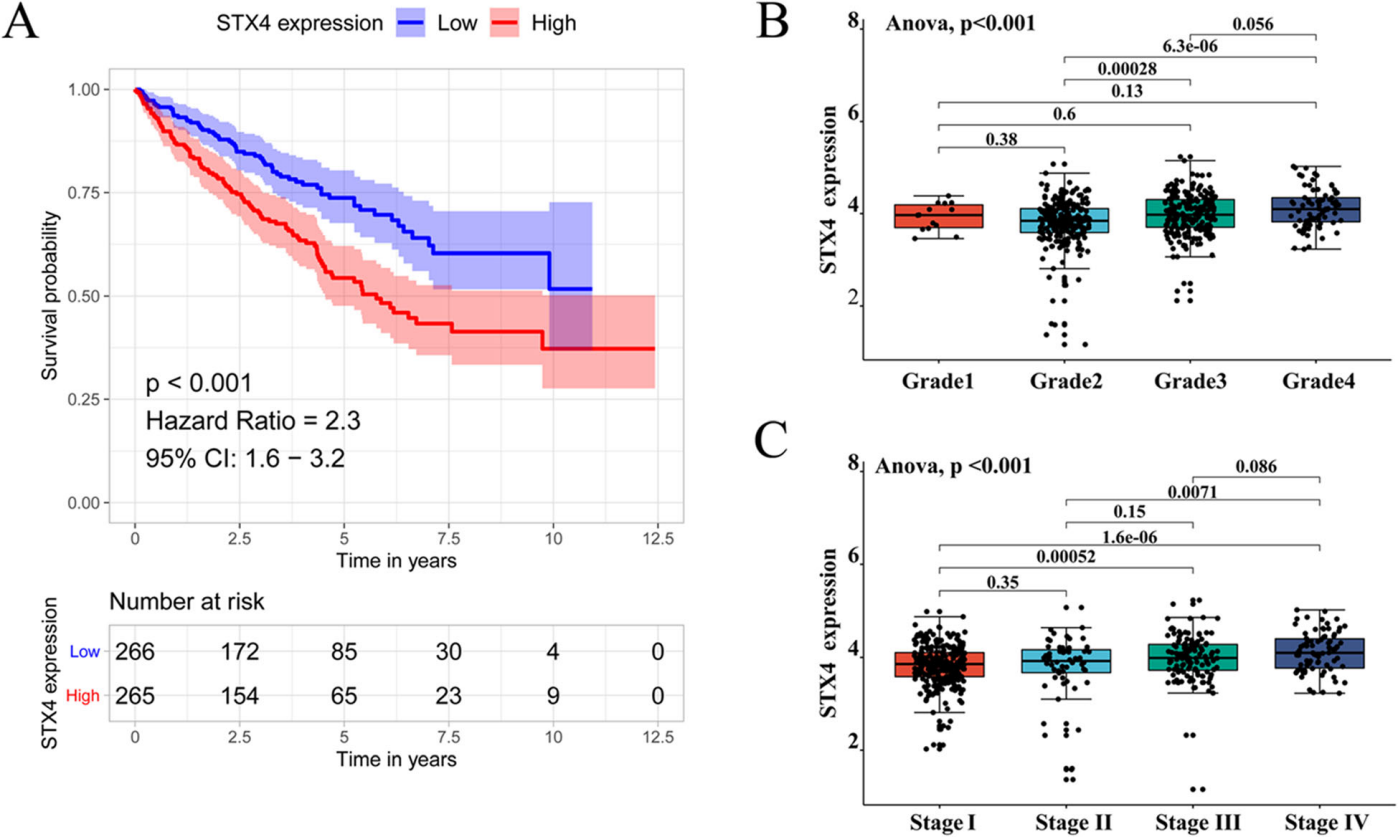
Prognosis and gene alteration of STX4 in KIRC. (**A**) STX4 up-regulation was significantly correlated with poorer overall survival in KIRC. (**B**) STX4 expression significantly elevated with increasing histological grade. (**C**) STX4 expression significantly elevated with advanced AJCC stages. KIRC, kidney renal clear cell carcinoma; AJCC, American Joint Committee on Cancer

Then we investigated the prognostic value of STX4 in KIRC. According to the median expression level of STX4, 531 patients with KIRC with survival information were divided into the high-STX4 and low-STX4 expression groups. As shown by the Kaplan–Meier (KM) survival analysis curve, there was a close relationship between the expression of STX4 and the survival of KIRC patients that the high expression of STX4 caused poor OS (HR = 2.3, *P* <  0.001, Fig. 2A). We next investigated the relationship between STX4 expression and the clinicopathological characteristics of KIRC. Analysis results showed that the STX4 expression level was significantly related to several clinicopathological features of KIRC, including cancer status (*P* = 0.003), histological grade (*P* <  0.001), tumor size (T stage, *P* <  0.001), distant metastasis (M stage, *P* <  0.001), and American Joint Committee on Cancer (AJCC) stage (*P* <  0.001) (Table 1). As shown in Fig. 2B, STX4 expression in KIRC tissues was significantly correlated with pathological grade (*P* <  0.001). Figure 2C showed the relationship between STX4 expression and different clinical stages, and the results suggested that STX4 expression was positively correlated with advanced clinical stage (*P* <  0.001).

To further validate the results, we explored the expression of STX4 in pan-cancer data (32 other cancer types) from TCGA as an internal validation. The “high” and “low” subgroups were always defined based on the mean expression value of STX4 in pan-cancer. According to the result of KM survival analysis, it is interesting that STX4 also played a prognostic role in cervical squamous cell carcinoma and endocervical adenocarcinoma, skin cutaneous melanoma, and uveal melanoma (Table 2). These results suggest that STX4 functions as a tumor promoter in KIRC.

**Table 2:** Kaplan-Meier survival analysis for STX4 in pan-cancer (33 types of cancer from TCGA)

Abbreviation	Detail	Log-rank test ***p*** value
ACC	Adrenocortical carcinoma	0.277
BLCA	Bladder Urothelial Carcinoma	0.702
BRCA	Breast invasive carcinoma	0.954
CESC	Cervical squamous cell carcinoma and endocervical adenocarcinoma	0.009
CHOL	Cholangio carcinoma	0.675
COAD	Colon adenocarcinoma	0.618
DLBC	Lymphoid Neoplasm Diffuse Large B-cell Lymphoma	0.629
ESCA	Esophageal carcinoma	0.309
GBM	Glioblastoma multiforme	0.098
HNSC	Head and Neck squamous cell carcinoma	0.438
KICH	Kidney Chromophobe	0.133
KIRC	Kidney renal clear cell carcinoma	0.000
KIRP	Kidney renal papillary cell carcinoma	0.573
LAML	Acute Myeloid Leukemia	0.185
LGG	Brain Lower Grade Glioma	0.888
LIHC	Liver hepatocellular carcinoma	0.102
LUAD	Lung adenocarcinoma	0.801
LUSC	Lung squamous cell carcinoma	0.936
MESO	Mesothelioma	0.319
OV	Ovarian serous cystadenocarcinoma	0.943
PAAD	Pancreatic adenocarcinoma	0.733
PCPG	Pheochromocytoma and Paraganglioma	0.453
PRAD	Prostate adenocarcinoma	0.161
READ	Rectum adenocarcinoma	0.766
SARC	Sarcoma	0.442
SKCM	Skin Cutaneous Melanoma	0.026
STAD	Stomach adenocarcinoma	0.155
TGCT	Testicular Germ Cell Tumors	0.315
THCA	Thyroid carcinoma	0.445
THYM	Thymoma	0.656
UCEC	Uterine Corpus Endometrial Carcinoma	0.832
UCS	Uterine Carcinosarcoma	0.248
UVM	Uveal Melanoma	0.003

### Independent prognostic analysis of STX4 in KIRC

**Fig. 3:**
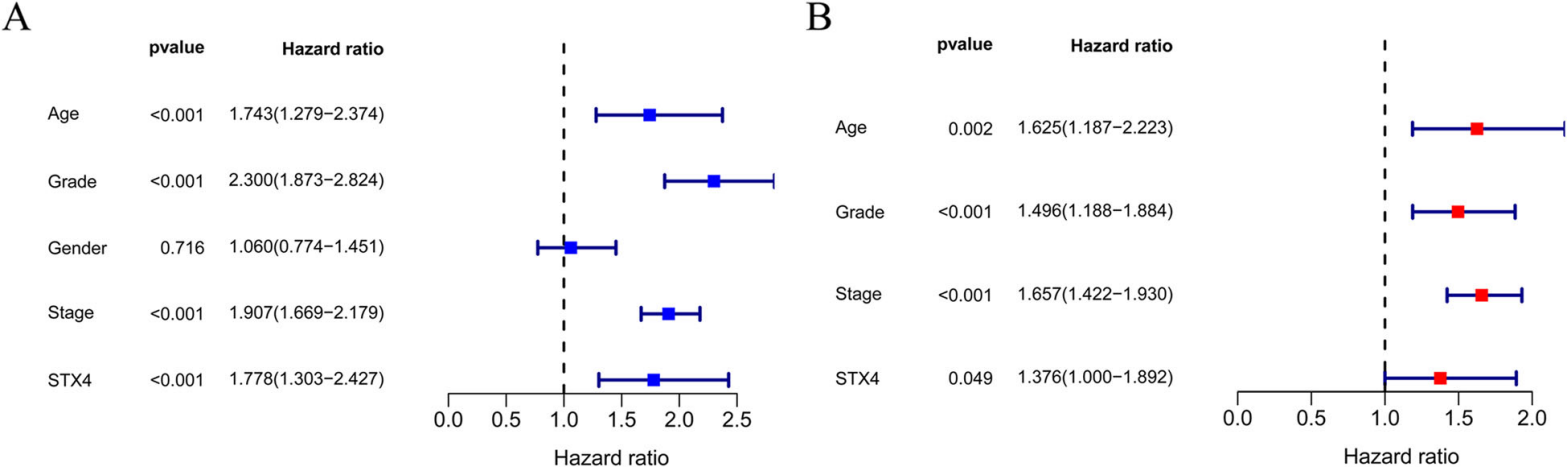
Forest plot of Cox regression analysis in KIRC. (**A**) Forest plot of univariate Cox regression analysis. (**B**) Forest plot of multivariate Cox regression analysis

On the basis of KM survival analysis curve showing that KIRC patients with up-expression of STX4 had obviously poor OS, univariate and multivariate Cox regression analyses were used to further explored whether STX4 had an independent prognostic value in KIRC. The results showed that there was a significantly prognostic difference between the high-STX4 and low-STX4 expression groups in both univariate (HR, 1.743; 95%CI, 1.279–2.374; *P* < 0.001) and multivariate (HR, 1.625; 95%CI, 1.187–2.223; *P* = 0.002) Cox regression analyses, which suggested that STX4 was an independent prognostic factor in KIRC (Fig. 3A, B).

### Functional and pathway enrichment analyses

GO functional enrichment analysis and KEGG analysis for STX4 were performed to investigate the molecular function and biological process of STX4. GO functional enrichment analysis for proteins interacting with STX4 demonstrated that the molecular functions cover protein binding processes, protein transport processes, and endosomal transport processes, autophagosome, and ubiquitin binding (Table 3). KEGG analysis revealed that the endocytosis, lysosome, and proteasome were enriched (Table 4). Meanwhile, mTOR signaling pathway, NOD-like receptor signaling pathway, and SNARE interactions in vesicular transport were also enriched (Table 4).

**Table 3:** GO function analysis for proteins interacting with STX4

Pathway description	Pathway ID	*P*-Value
protein binding	GO:0005515	8.33E-89
cytosol	GO:0005829	2.27E-32
cytoplasm	GO:0005737	4.31E-30
identical protein binding	GO:0042802	4.51E-10
spliceosomal complex	GO:0005681	1.20E-08
molecular_function	GO:0003674	5.40E-06
plasma membrane	GO:0005886	8.27E-06
membrane	GO:0016020	8.51E-06
protein transport	GO:0015031	9.29E-06
protein kinase binding	GO:0019901	1.06E-05
intracellular signal transduction	GO:0035556	1.50E-05
protein phosphorylation	GO:0006468	5.34E-05
tumor necrosis factor-mediated signaling pathway	GO:0033209	7.14E-05
intracellular membrane-bounded organelle	GO:0043231	0.000155436
transcription export complex	GO:0000346	0.000206682
apoptotic process	GO:0006915	0.000229098
endosomal transport	GO:0016197	0.000322781
intracellular protein transport	GO:0006886	0.000350599
U2-type prespliceosome	GO:0071004	0.000478136
autophagosome	GO:0005776	0.000499235
microtubule organizing center	GO:0005815	0.000522493
endomembrane system	GO:0012505	0.000786406
signal transduction	GO:0007165	0.000824551
ubiquitin binding	GO:0043130	0.000930267

**Table 4:** KEGG function analysis for proteins interacting with STX4

Pathway description	description ID	*P*-Value
Spliceosome	hsa03040	1.00E-06
Endocytosis	hsa04144	0.000147263
Choline metabolism in cancer	hsa05231	0.000232401
mRNA surveillance pathway	hsa03015	0.000322781
Homologous recombination	hsa03440	0.000405621
Glycerophospholipid metabolism	hsa00564	0.000573028
Lysosome	hsa04142	0.000672647
RNA transport	hsa03013	0.000864392
Necroptosis	hsa04217	0.001676903
NOD-like receptor signaling pathway	hsa04621	0.001929387
C-type lectin receptor signaling pathway	hsa04625	0.002830637
Epstein-Barr virus infection	hsa05169	0.00314403
GnRH signaling pathway	hsa04912	0.003202064
Phospholipase D signaling pathway	hsa04072	0.003763014
VEGF signaling pathway	hsa04370	0.003815766
mTOR signaling pathway	hsa04150	0.005035986
Non-small cell lung cancer	hsa05223	0.007316192
Base excision repair	hsa03410	0.008263833
Pathways in cancer	hsa05200	0.008968622
Proteasome	hsa03050	0.009338017
SNARE interactions in vesicular transport	hsa04130	0.009352469

## Discussion

KIRC was one of the highest incidence subtypes of the RCC with poor prognosis. Due to limited predictors assessing the risk because of tumor heterogeneity, a part of KIRC patients with poor prognosis might be miss aggressive treatment due to a delay in diagnosis, which would decrease the survival rate of patients with KIRC to some extent. Hence, it is crucial to identify new specific prognosis predictors for KIRC.

STX4 is one of the SNAREs proteins and is involved in cell invadopodium formation and tumor cell invasion [9, 10]. Research has shown that STX4 also plays an important role in several cancers. A research had identified the exocytosis mediator proteins STX4 in the peripheral blood neutrophils of patients with chronic myeloid leukemia early in 2004 [12]. Breast cancer showed the strongest correlation with the expression of STX4, the latter was associated with reduced patient survival in high expression [13]. Extracellular STX4 triggers the differentiation program in teratocarcinoma F9 cells that impacts cell adhesion properties [14]. It still had not study reveal the effect of STX4 in KIRC. Here, we screened out available datasets associated with KIRC from public databases to confirm the function of STX4 on the oncoming, progression, and prognosis of KIRC. In this study, STX4 up-regulation was significantly associated with unfavorable clinicopathological features in KIRC, such as higher histological grade, larger tumor size, distant metastasis, and advanced AJCC stage. KM survival analysis curve showed that STX4 expression had maintained a high level with a poor OS. Furthermore, univariate and multivariate Cox regression analyses confirmed that STX4 played an independent prognostic role in KIRC. The qRT-PCR results from 10 paired of KIRC and normal real-world samples further confirmed the up-regulation of STX4 in KIRC. This suggested that STX4 could be identified as a potential prognostic biomarker in KIRC.

We used data of 32 other types of cancer from TCGA to validate the aforementioned results. As the pan-cancer analysis result shown that survival differences of STX4 existed in several types of cancer. All results were consistent and suggested that STX4 may serve as a tumor promoter in KIRC.

Further research into how STX4 influences patients’ survival via GO functional enrichment analysis and KEGG analysis. These analyses demonstrated that the molecular functions of STX4 cover protein binding processes, protein transport processes, endosomal transport processes, and endocytosis, which suggest that STX4 influences the endo/exocytosis of the tumor. The fusion of secretory vesicles and subsequent protein exocytosis are the important mechanism of cancer cells metastasis [7]. Alterations of endo/exocytic proteins have long been associated with malignant transformation, and genes encoding membrane trafficking proteins have been identified as bona fide drivers of tumorigenesis [15]. Results had demonstrated that STX4 defines a domain for activity-dependent exocytosis in dendritic spines [16, 17] STX4 mediated trafficking of MT1-MMP during invadopodium formation and tumor cell invasion [10, 18]. Autophagosome, lysosome, mTOR signaling pathway, and NOD-like receptor signaling pathway were also enriched by GO and KEGG analyses. Autophagy is a lysosomal-dependent pathway for intracellular degradation, leading to the basal turnover of cell components and providing energy and macromolecular precursors. Autophagy has opposing, context-dependent roles in cancer, and interventions to both stimulate and inhibit autophagy have been proposed as cancer therapies [19]. In addition, mTOR signaling pathway and NOD-like receptor signaling pathway play a crucial role in regulating autophagy [20–22]. STX2 to block STX3- and STX4-mediated fusion of zymogen granules with the plasma membrane and exocytosis and prevent binding of ATG16L1 to clathrin, which contributes to induction of autophagy [23]. Thus, we speculated that STX4 may promote the tumor progression and influence the prognosis of KIRC by regulating endo/exocytosis, and autophagy.

This study has several limitations, although it is the first to discover the potential prognostic value of STX4 in KIRC. First, although differential STX4 expression was detected between tumor and normal real-world samples, the prognostic implication of this finding has not been demonstrated. Second, only transcriptomics expression of STX4 with clinical data was analyzed to predict OS in this study. Third, the underlying mechanisms of STX4 in KIRC remain unclear, only function enrichment analyses were performed. Therefore, additional data and samples are necessary to confirm the results of this study. Future research is required to explore the detailed molecular mechanism of STX4 in KIRC.

In conclusion, this study demonstrated that STX4 is a key survival-associated marker in KIRC. With a potential role in endo/exocytosis, STX4 may be a novel therapeutic target in patients with KIRC.

## Data Availability

The datasets generated and/or analysed during the current study are available in the [The Cancer Genome Atlas (TCGA) through the University of California Santa Cruz Xena database platform] repository, (https://xena.ucsc.edu/).

## Abbreviations

KIRC: Kidney renal clear cell carcinoma
OS: Overall survival
TCGA: The Cancer Genome Atlas
PresSTIGE: Predicting Specific Tissue Interactions of Genes and Enhancers
qRT-PCR: Quantitative reverse transcription polymerase chain reaction
GAPDH: glyceraldehyde 3-phosphate dehydrogenase
GO: Gene Ontology
KEGG: Kyoto Encyclopedia of Genes and Genomes
AJCC: American Joint Committee on Cancer

## References

[1] Ljungberg B, Bensalah K, Canfield S, Dabestani S, Hofmann F, Hora M (2015). EAU guidelines on renal cell carcinoma: 2014 update. Eur Urol.

[2] Siegel RL, Miller KD, Jemal A (2019). Cancer statistics, 2019. CA Cancer J Clin.

[3] Gray RE, Harris GT (2019). Renal cell carcinoma: diagnosis and management. Am Fam Physician.

[4] Hsieh JJ, Purdue MP, Signoretti S, Swanton C, Albiges L, Schmidinger M (2017). Renal cell carcinoma. Nat Rev Dis Primers.

[5] Mitsui Y, Shiina H, Kato T, Maekawa S, Hashimoto Y, Shiina M (2017). Versican promotes tumor progression, metastasis and predicts poor prognosis in renal carcinoma. Mol Cancer Res.

[6] Motzer RJ, Jonasch E, Agarwal N, Beard C, Bhayani S, Bolger GB (2015). National comprehensive cancer network. Kidney cancer, version 3. 2015. J Natl Compr Cancer Netw.

[7] Stephens DC, Harris DA (2020). Organizing 'Elements': facilitating exocytosis and promoting metastasis. Trends Cancer.

[8] Chen YA, Scheller RH (2001). SNARE-mediated membrane fusion. Nat Rev Mol Cell Biol.

[9] Miyata T, Ohnishi H, Suzuki J, Yoshikumi Y, Ohno H, Mashima H (2004). Involvement of syntaxin 4 in the transport of membrane-type 1 matrix metalloproteinase to the plasma membrane in human gastric epithelial cells. Biochem Biophys Res Commun.

[10] Brasher MI, Martynowicz DM, Grafinger OR, Hucik A, Shanks-Skinner E, Uniacke J (2017). Interaction of Munc18c and syntaxin4 facilitates invadopodium formation and extracellular matrix invasion of tumor cells. J Biol Chem.

[11] Xie C, Mao X, Huang J, Ding Y, Wu J, Dong S (2011). KOBAS 2.0: a web server for annotation and identification of enriched pathways and diseases. Nucleic Acids Res.

[12] Nuyanzina VA, Nabokina SM (2004). Identification of exocytosis mediator proteins in peripheral blood neutrophils of patients with chronic myeloid leukemia. Bull Exp Biol Med.

[13] Althubiti M, Lezina L, Carrera S, Jukes-Jones R, Giblett SM, Antonov A (2014). Characterization of novel markers of senescence and their prognostic potential in cancer. Cell Death Dis.

[14] Hagiwara N, Kadono N, Miyazaki T, Maekubo K, Hirai Y (2013). Extracellular syntaxin4 triggers the differentiation program in teratocarcinoma F9 cells that impacts cell adhesion properties. Cell Tissue Res.

[15] Lanzetti L, Di Fiore PP (2017). Behind the scenes: Endo/exocytosis in the Acquisition of Metastatic Traits. Cancer Res.

[16] Kennedy MJ, Davison IG, Robinson CG, Ehlers MD (2010). Syntaxin-4 defines a domain for activity-dependent exocytosis in dendritic spines. Cell..

[17] Mohanasundaram P, Shanmugam MM (2010). Role of syntaxin 4 in activity-dependent exocytosis and synaptic plasticity in hippocampal neurons. Sci Signal.

[18] Williams KC, McNeilly RE, Coppolino MG (2014). SNAP23, Syntaxin4, and vesicle-associated membrane protein 7 (VAMP7) mediate trafficking of membrane type 1-matrix metalloproteinase (MT1-MMP) during invadopodium formation and tumor cell invasion. Mol Biol Cell.

[19] Levy JMM, Towers CG, Thorburn A (2017). Targeting autophagy in cancer. Nat Rev Cancer.

[20] Kim YC, Guan KL (2015). mTOR: a pharmacologic target for autophagy regulation. J Clin Invest.

[21] Kim YK, Shin JS, Nahm MH (2016). NOD-like receptors in infection, immunity, and diseases. Yonsei Med J.

[22] Travassos LH, Carneiro LA, Ramjeet M, Hussey S, Kim YG, Magalhães JG (2010). Nod1 and Nod2 direct autophagy by recruiting ATG16L1 to the plasma membrane at the site of bacterial entry. Nat Immun.

[23] Dolai S, Liang T, Orabi AI, Holmyard D, Xie L, Greitzer-Antes D (2018). Pancreatitis-induced depletion of Syntaxin 2 promotes autophagy and increases Basolateral exocytosis. Gastroenterology.

